# Separation and conversion dynamics of nuclear-spin isomers of gaseous methanol

**DOI:** 10.1038/ncomms7877

**Published:** 2015-04-16

**Authors:** Zhen-Dong Sun, Meihua Ge, Yujun Zheng

**Affiliations:** 1 School of Physics, Shandong University, 27 Shanda Nanlu, Jinan, Shandong 250100, China; 2 Department of Physics, University of Toyama, Toyama 930-8555, Japan

## Abstract

All symmetrical molecules with non-zero nuclear spin exist in nature as nuclear-spin isomers (NSIs). However, owing to the lack of experimental information, knowledge is rare about interconversions of NSIs of gaseous molecules with torsional symmetry. Here we report our separation and conversion observations on NSI-torsion-specific transition systems of gaseous methanol from a light-induced drift experiment involving partially spatial separation of the ortho and para isomers. We find that vibrationally excited molecules of the methanol spin isomer have a smaller collision cross-section than their ground-state counterparts. Interconversion of the enriched ortho isomer with the para isomer, which is generally considered improbable, has been quantitatively studied by sensitive detections of the spectral intensities. Rather counterintuitively, this reveals that the interconversion is inhibited with increasing pressure. Our results suggest that the spin conversion mechanism in methanol is via a quantum relaxation process with the quantum Zeno effect induced by molecular collisions.

## Abstract (web summary)

The conversion dynamics of nuclear-spin isomers has only been observed for a small number of molecules, generally with rotational symmetry. Here, the authors observe the separation of nuclear-spin isomers of gaseous methanol and show a decreased interconversion at higher pressures.

## Graphical abstract

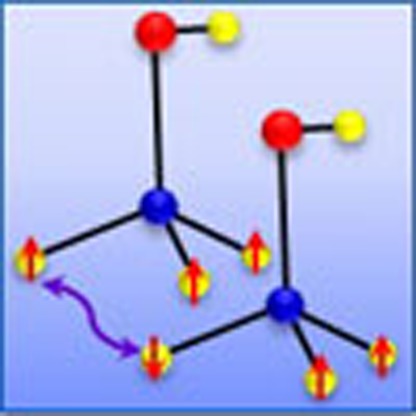

In quantum mechanics^1^, nuclear-spin isomers (NSIs) are different molecular species and each of the NSIs of a molecule can be spectroscopically identified by different classes of quantum numbers in the ground-state levels of transitions of the molecule. However, despite the fact that continuous studies of NSIs of molecules began as early as the first separation and conversion of ortho- and para-H_2_ in 1929 (ref. 2), yet very little is known about the interconversion dynamics of NSIs of gaseous molecules with torsional symmetry. Although many symmetrical molecules have distinct NSIs, to date, separation and conversion dynamics have been successfully studied for just a few gaseous molecules, including H_2_, CH_3_F (ref. 3), ^13^CH_3_F (refs 4−6, 5, 6), H_2_CO (ref. 7), ^13^CCH_4_ (ref. 8), C_2_H_4_ (refs 9, 10) and H_2_O (refs 11, 12). All the NSIs of these molecules are identified by rotational quantum numbers. Among these molecules, only the NSIs of CH_3_F, ^13^CH_3_F, ^13^CCH_4_ and C_2_H_4_ have been separated at ambient temperature in the gas phase by the method of light-induced drift^13^ (LID) and the nuclear-spin conversion mechanisms of them have been explained by the model of quantum relaxation^14^. In astrophysics, measurements of the ortho-to-para ratios of any interstellar molecules can give important molecular formation information^15^,^16^,^17^,^18^,^19^. However, despite the fact that methanol (CH_3_OH) is one of the most abundant interstellar molecules^20^,^21^, so far this has not been achieved for CH_3_OH.

CH_3_OH is the simplest alcohol, having a C_3V_(M) symmetry point group and displaying internal rotation or torsion of the CH_3_ group with respect to the OH group. It is from the three spin–1/2 hydrogen nuclei of this CH_3_ group that the ortho (total nuclear spin quantum number *I*=3/2) and para (*I*=1/2) nuclear spin isomers are formed, which are distinguished respectively by the symmetry quantum numbers *σ*=0 and ±1. Here, each *σ* value corresponds to a torsional-symmetry species of the C_3_ group *A* (*σ*=0) and *E* (*σ*=±1), and combines with the *A*- and *E*-species in the spin part of the molecular wavefunction. Although CH_3_OH is one of the simplest asymmetric-top molecules with one hindered large-amplitude torsion, its spectrum is quite complicated due to strong coupling of torsion with other vibrational modes and has been extensively studied for many years as a prototype nonrigid molecule in molecular physics^22^,^23^,^24^. To date, however, little experimental information about the specific physical properties of two spin-isomer molecules, such as their stability and how the collision cross-section (CCS) of the ortho- or para-isomer molecules varies on excitation, has been obtained for gaseous CH_3_OH. No population transfers between the *A*- and *E*-levels have ever been observed and no evidence of weak intramolecular hyperfine interactions producing the ortho–para state mixing in CH_3_OH has been found. Thus, to seek the possibility of separation of the ortho and para isomers by the LID method and to provide experimental evidence and explanation of mechanisms to the interconversion between these two spin isomers, we performed separation and conversion observations on CH_3_OH in the gas phase.

In this work, we report experimental results on performing nuclear spin enrichment and nuclear spin conversion in gaseous CH_3_OH. The enrichment is performed by the LID method and shows an unusual LID effect, which has the opposite direction of the drift of the NSIs to that observed in the LID effect for other molecular NSIs. We demonstrate the observation of the decreasing of nuclear spin conversion rate with increasing pressure and explain this novel and interesting phenomenon in a free molecule by the model of quantum relaxation. The experiment has been performed several times with different pressures for the ortho and para isomers of CH_3_OH independently. Importantly, in contrast to the molecular NSIs studied before, the mixing between ortho and para states involves torsional wavefunctions and the intramolecular torsion is involved in the nuclear spin conversion in CH_3_OH. In this work we provide experimental evidence for the existence of the weak nuclear-spin-dependent intramolecular hyperfine interactions in gaseous CH_3_OH.

## Results

### Experimental schemes

**Figure 1:**
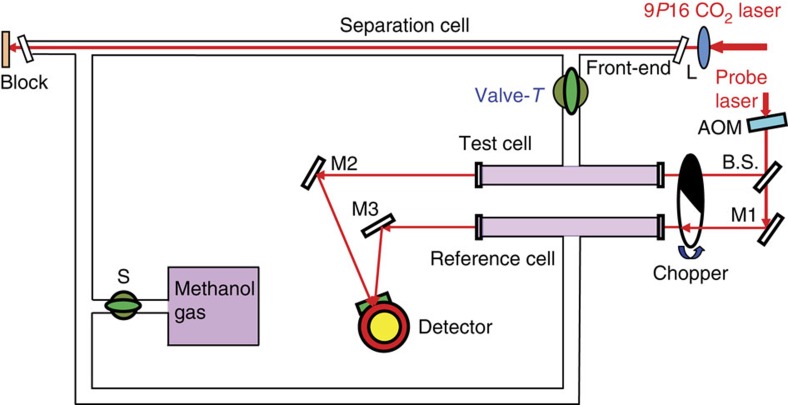
Schematic of the experimental setup. L, ZnSe lens of focal length 25 cm; AOM, acousto-optic modulator; M1–M3, mirrors; BS, beam splitter in ratio of 1:1; S, glass valve; Detector, the liquid-N_2_-cooled HgCdTe detector. The probe CO_2_ laser line was set at 9*P*12 in Case 1 and at 9*P*42 in Case 2, respectively.

**Figure 2:**
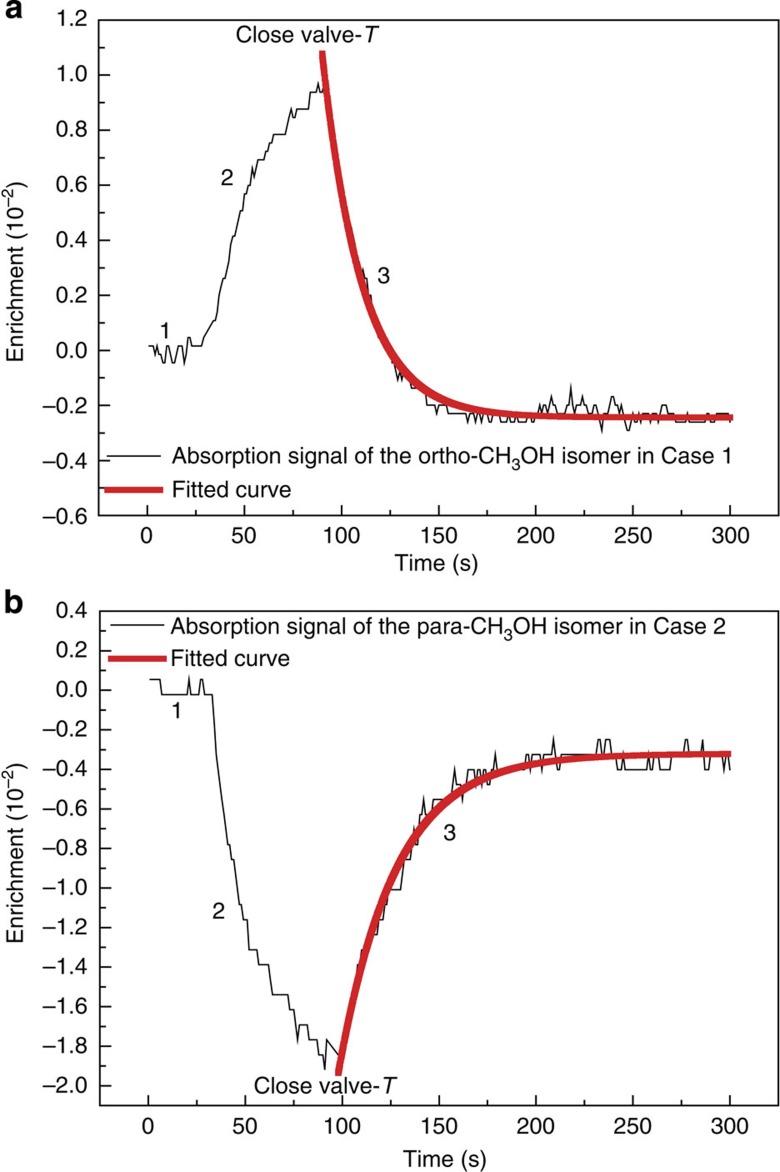
Recorded differential absorption signals. (**a**) By probing laser frequency of *f*_0_(9*P*12)+100 MHz for the ortho isomer at 0.80 Torr. (**b**) By probing laser frequency of *f*_0_(9*P*42)−100 MHz for the para isomer at 0.76 Torr. The traces in the first period are the zero-difference baselines. After 1 min separation and at the 90th second, the enrichment of the ortho isomer is 0.97 (±0.11) × 10^−2^ (**a**) and the depletion of the para isomer is 1.83 (±0.15) × 10^−2^ (**b**). The traces in the third period show the isomer conversions after closing the valve-*T* and are exponentially fitted with smooth curves. The negative signal offsets from the zero-difference baselines are caused by the net wall adsorptions of the enriched ortho isomer and the para isomer in the test cell during the separation stage.

A schematic of our experimental setup is shown in Fig. 1, which has been described in detail previously^9^. The experimental schemes for CH_3_OH are shown in Table 1. The 9*P*16 CO_2_ laser line was selected to create a non-equilibrium isomeric mixture near the front end of the 1-m-long separation cell and in the test cell through the valve-*T* by using the effect of LID. The centre frequency of the 9*P*16 CO_2_ laser line *f*_0_(9*P*16), from the reported observations and spectral assignments in high-resolution Fourier-transform spectra of CH_3_OH (refs 22, 23), was calculated to be ∼67 MHz below the centre frequency of the *R*(*σ υ*_t_ *K*, *J*)=*R*(*A* 0 0, 10) line, where *υ*_t_ is the torsional quantum number, and *J* and *K* are quantum numbers of the rotational angular momentum and its projection along the axis of the CH_3_ group, respectively. This laser line was thus tuned into the Doppler-broadened absorption profile of the *R*(*A* 0 0, 10) line for separating the ortho isomer from the para isomer in the experiment. Another frequency-stabilized CO_2_ laser was used for detection of the ortho (Case 1) or para isomer (Case 2) concentration with shifting its centre frequency of 100 MHz by an infrared acousto-optic modulator. By using an optical chopper, two probe laser beams from the second laser were aligned in anti-phase simultaneously passing through the test cell and the reference cell for sensitive detections of the differential spectral intensities. As such, the output of the detector recombined from these detection cells directly reflects the non-equilibrium concentrations of spin isomers in the test cell. Here, the reference cell is of the same size as the test cell and kept at the equilibrium composition. Accordingly, the LID effects and the decay curves arising from subsequent population transfer between the ortho and para isomers after closing the valve-*T* were observed by employing a three-period signal recording method (see Methods). Typical recorded differential absorption signals are shown in Fig. 2a for Case 1, with the *R*(*A* 0 5, 13) line detected by the probe laser frequency at *f*_0_(9*P*12)+100 MHz, and in Fig. 2b for Case 2, with the *P*(*E* 0 1, 5) line detected by the probe laser frequency at *f*_0_(9*P*42)−100 MHz.

The first signal period (Stage 1) represents the zero-difference baseline corresponding to the equilibrium level of the ortho isomer or the para isomer in the natural abundance ratio. It was recorded starting 3 min late after filling the sample cells with CH_3_OH, to allow for adsorption of CH_3_OH on the cell walls.

### Observation of the unusual LID effect

In Stage 2, the 9*P*16 separation laser line was tuned about 20 MHz above *f*_0_(9*P*16) by adjusting the laser cavity length and the 16 W output power of it was focused into the separation cell from the front end for 1 min to initiate isomer separation. Here, because of the Doppler effect only a specific velocity subgroup of ortho molecules in the red wing of the *R*(*A* 0 0, 10) line moving towards the laser beam was selectively excited, with the other non-absorbing molecules acting as a buffer gas. If these excited ortho-CH_3_OH molecules were to have a larger CCS than the ground-state molecules, as previously observed in what we will call the usual LID on NSIs of CH_3_F, ^13^CH_3_F, ^13^CCH_4_ and C_2_H_4_, then their mean free path would be shorter than that of the ground-state molecules and they would thereby be slowed down by collisions with the buffer gas. Unexcited molecules in the ground state, however, would keep moving with the normal speed along the laser beam. This would produce a net drift of the ortho-CH_3_OH molecules with respect to the buffer gas along the laser direction and result in depletion of the ortho isomer and relative enrichment of the para isomer at the front end of the separation cell. However, we observed the opposite effect, namely an enrichment of the ortho isomer (Fig. 2a) and a depletion of the para isomer (Fig. 2b) in the test cell revealed clearly by the probing resonances in Cases 1 and 2. This shows that the ortho-CH_3_OH molecules actually drift towards the laser beam in the separation cell and indicates a diminution in CCS for the excited ortho molecules relative to their ground-state counterparts. We take such an LID produced by laser-excited isomer molecules having a smaller CCS than the ground-state molecules to be an unusual LID, as the excited molecules usually have a larger CCS than the ground-state molecules^25^. Similar absorption signals to Fig. 2a were also observed for the *R*(*A* 0 0, 10) line itself by using the acousto-optic modulator to shift the probe laser frequency 100 MHz above *f*_0_(9*P*16), to probe a different velocity subgroup of the ortho molecules. Besides this, two additional observations were made to double check the self-consistency of our unusual LID results using the same probing resonance as in Case 1. First, we inverted the direction of the separation laser beam so that it passed out of the front end of the separation cell. In this situation, population depletion of the ortho isomer in Stage 2 was observed with opposite sign to that in Fig. 2a, confirming the unusual LID results and also revealing that the NSI separation can be sensitively controlled by the laser. Second, we pumped the *Q*(*E* 0 8, 16) para line, which is calculated to lie 31 MHz higher than *f*_0_(9*P*36). When we tuned the separation laser frequency to about *f*_0_(9*P*36)+15 MHz in resonance with the *Q*(*E* 0 8, 16) line, similar absorption signals to those in Fig. 2b were observed, indicating the same unusual LID effects as those in Fig. 2b. This supports our ortho results and confirms that the CCS of excited molecules is smaller than that of unexcited ground-state molecules for the para species as well.

### Explanation of the interconversion of ortho and para isomers

In ordinary gas-phase molecular spectroscopy, molecular population transfers and transitions between the *A* and *E* two different torsional-symmetry species are generally considered improbable, because they are forbidden by the electric-dipole selection rules of the molecule and their spectral intensities would be extremely weak; therefore, such transfers and transitions have never been directly seen for gaseous CH_3_OH. However, we have observed the population variations of the ortho and para isomers, as seen from the third period (Stage 3) of the signals in Fig. 2. The spectral intensity of the ortho isomer decreases (Fig. 2a) and that of the para isomer increases (Fig. 2b) towards their equilibrium levels. This general phenomenon and the offset between the new equilibrium level and the zero-difference baseline can be qualitatively explained as follows. In the initial seconds of Stage 2, as the hot ortho-CH_3_OH molecules from the separation cell quickly enter and the para-CH_3_OH molecules fast exit the test cell through the valve-*T* due to the unusual LID effect, and also because the inner surface of the test cell (mainly the part opposite to the sidearm of the valve-*T*) gets in their way of such rapid influxes of the hot ortho-CH_3_OH molecules, there are head-on collisions and therefore enhanced net adsorptions of the ortho-CH_3_OH molecules on the wall of the test cell, leading to the degree of enrichment of the ortho isomer to be smaller than the degree of depletion of the para isomer. However, as the isomeric conversion also takes place simultaneously and competes against the isomeric separation in Stage 2, the enrichment and the enhanced adsorption gradually saturate as the separation proceeds and reach saturations when the test cell is isolated by closing the valve-*T*. Hence, relative to the zero-difference baseline, a reduced equilibrium level of the isomeric conversion exists and remains almost unchanged in the subsequent isomeric decay stage over the time range studied. In the isolated test cell, apart from the surface-molecule collisions, there are also molecule–molecule collisions within the bulk gas, through which the ortho and para molecules can move up and down within their own manifolds of states. There are certain particular pairs of ortho and para states whose energy gaps are so small that they are coupled and strongly mixed by the weak nuclear-spin-dependent intramolecular hyperfine interactions of CH_3_OH, allowing molecules arriving in these doorway states to freely move between ortho and para manifolds during the mixing^26^. However, the time evolution of this state mixing is interrupted by the next collision, following which those freely moving isomer molecules can end up either in the same or a different spin state than that in which they started through a quantum relaxation process^14^.

### Analysis of the quantum relaxation process of CH_3_OH isomers

**Figure 3:**
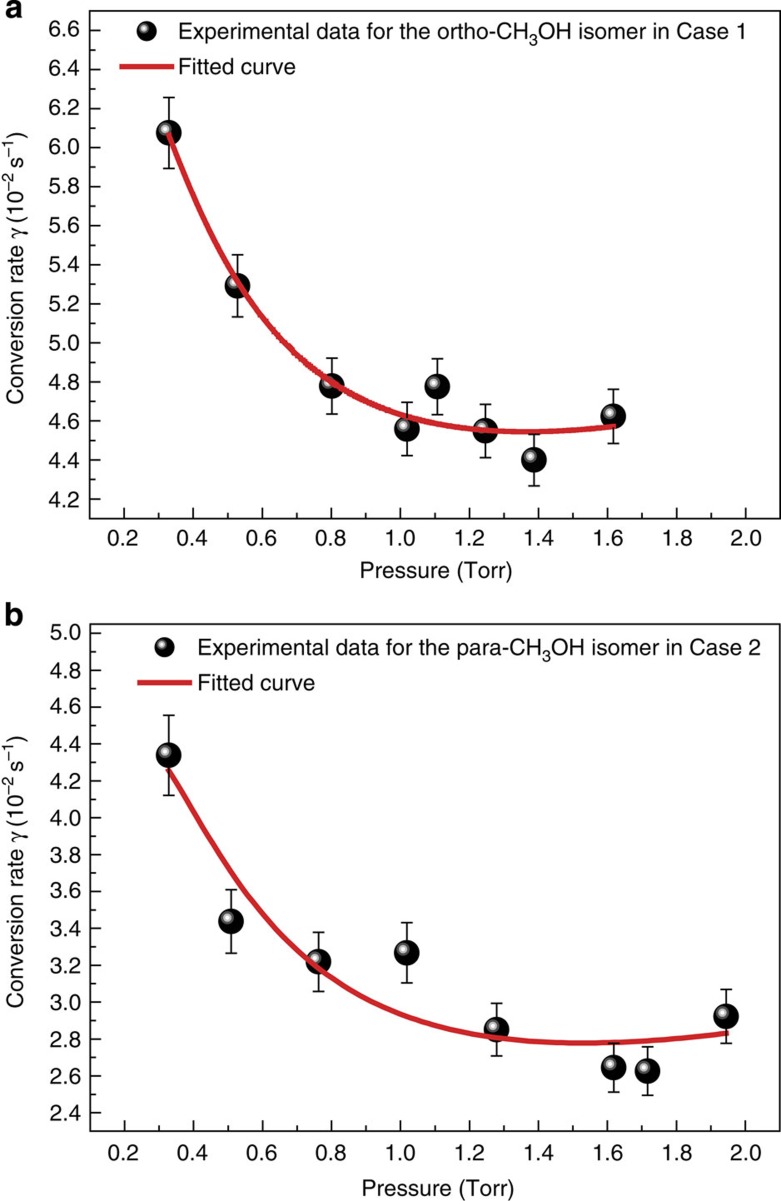
The observed (solid dots) and fitted (smooth curves) conversion rates as a function of pressure. (**a**) At probe laser frequency of *f*_0_(9*P*12)+100 MHz for the ortho-CH_3_OH isomer. (**b**) At probe laser frequency of *f*_0_(9*P*42)−100 MHz for the para-CH_3_OH isomer.

A general quantitative description of the quantum relaxation process of ortho–para isomer conversion has shown that the relaxation of a non-equilibrium concentration of the ortho or para isomer at low pressures is an exponential decay with the conversion rate^14^


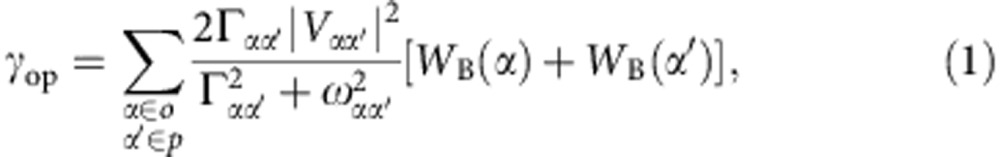


where *W*_B_(*α*) and *W*_B_(*α*′) are the Boltzmann factors of the *α* (ortho) and *α*′ (para) states, respectively, *ω*_*αα*′_/2*π* is the energy gap in a unit of frequency, *V*_*αα*′_ is the matrix element of the magnetic interaction that creates a coherence between the α and *α*′ states and Γ_*αα*′_ is the decoherence rate of this coherence that is proportional to the gas pressure *p*, and the summation is over all pairs of ortho and para levels. As for the decay data of Fig. 2, the function *A* exp(−*γt*)+*B*, where *A* is the integrated intensity and *B* is the signal offset from the zero-difference baseline, was fitted by least squares to the third period of the recorded signal to give the solid smooth curve and the observed conversion rate *γ*. The determined *γ*-values are 4.86 (±0.09) × 10^−2^ s^−1^ (Fig. 2a) and 3.39 (±0.07) × 10^−2^ s^−1^ (Fig. 2b). We have repeated the conversion measurements over 180 times for Case 1 and over 200 times for Case 2 at various pressures. The mean values of the observed conversion rates (solid dots) are plotted as a function of pressure in Fig. 3. Contrary to the intuitive expectation that more rapid collisions of molecules would speed up the relaxation, it is seen in Fig. 3 that the measured conversion rate decreases quickly between 0.3 and 1 Torr, then varies slowly between 1 and 2 Torr. This behaviour of CH_3_OH is also in contrast to the previously observed linear pressure dependences of the conversion rates in NSIs of CH_3_F, ^13^CH_3_F, H_2_CO, ^13^CCH_4_ and C_2_H_4_. For these species, the energy gaps satisfy *ω*_*αα*′_>>Γ_*αα*′_ at low pressures; hence, from equation (1) one finds *γ*_op_∝Γ_*αα*′_∝*p*. For CH_3_OH, however, we found that there are a number of ortho–para level pairs obeying the selection rules |Δ*J*|≤2 and |Δ*K*|≤2, for which the energy gaps *ω*_*αα*′_/2*π* (see also Supplementary Table 1) are close to Γ_*αα*′_/2*π*=*Cp* for *p* in 0.3–2 Torr with *C*=20 MHz Torr^−1^ (refs 22, 27); thus, they are expected to contribute considerably to the conversion rates. Therefore, the expression *γ*≈*ap*/(*b*+*p*^2^)+*cp*+*d* (see Methods) is considered to better characterize the physical nature behind the spin conversion processes in our experiment and is fitted to the experimental data in Fig. 3 (smooth curves). Here, *b*=(*ω*/40*π*)^2^, *a* is a rate constant and *c* is the constant for a linear pressure term, terms *cp* and *ap*/(*b*+*p*^2^) stand for the rates of the spin conversions contributed from molecular population transfers via the level pairs with *ω*_*αα*′_>>Γ_*αα*′_ and the most closely near-degenerate pair with *ω*∼Γ_*αα*′_, respectively, the pressure-independent term *d* approximately expresses the induced rate of spin conversion by interactions of the enriched ortho and the depleted para molecules with the isomer-adsorbed wall of the test cell. From the fits, the obtained *d*-value is 2.56 (±0.54) × 10^−2^ s^−1^ for the ortho isomer in Case 1 and is 0.82 (±0.30) × 10^−2^ s^−1^ for the para isomer in Case 2 (see also Supplementary Note 1). To derive the contributions of intramolecular hyperfine interactions to the nuclear spin conversion rates of the CH_3_OH isomers, the obtained *d*-values were subtracted from the observed conversion rates and the corrected conversion rates were then fitted to the expression *γ*_op_≈*ap*/(*b*+*p*^2^)+*cp*. We see that the obtained ortho–para conversion parameters in Cases 1 and 2 in Table 1 agree well within the experimental errors. The results reveal that the interconversion between the ortho- and para-CH_3_OH isomers occurs in a quantum relaxation process, mainly through the strongly mixed near-degenerate ortho–para level pairs, and is naturally curtailed with increasing pressure. The latter is because the greater rate of molecular collisions reduces the interaction times available for evolution of the mixing between collisions and limits the efficiency of the state mixing. Such an inhibition of conversions between the ortho and para states with increasing pressures can be considered as an example of the quantum Zeno effect^28^ induced by collisions in a free molecule. A similar effect has also been observed for ^13^CH_3_F in an applied external electric field where the energy gaps between the interacting ortho and para substates of ^13^CH_3_F were narrowed via splitting of the levels^5^.

## Discussion

In our experiment, we have enriched the population of the ortho isomer using the 9*P*16 CO_2_ laser line. However, owing to the further isomeric adsorptions to the wall of the test cell in the separation process, the enrichment of ortho species in Case 1 is smaller than the depletion of para species in Case 2 at the beginning of the recorded decay curves in the nuclear-spin conversion processes. We have quantitatively accounted for our nuclear-spin conversion observations by the model of quantum relaxation and experimentally determined the spin-conversion rate *γ*; the latter for the ortho isomer in Case 1, owing to larger induced spin-conversion rate by the molecule–surface interactions is bigger than that for the para isomer in Case 2. Considering the contributions of molecular collisions to the interconversion of the spin isomers, our experimentally determined spin conversion rate *γ* can be taken as the sum of the spin conversion rate caused by molecule–molecule collisions *γ*_mm_ and by molecule–surface collision *γ*_ms_, that is, *γ*=*γ*_mm_+*γ*_ms_=*γ*_op_+*d*. Here, *γ*_ms_ is the parameter *d*, a pressure-independent term and it approximately expresses the induced-rate of spin conversion by the interactions of molecules with the wall of the test cell. Molecule–surface collisions may cause adsorptions and desorptions of the ortho and para isomers on the cell wall where the stuck ortho and para isomers may remain for different time periods. The inhomogeneous magnetic field within the part volume of the test cell may induce flips of spin isomers. Comparing with the para isomer, the enriched ortho isomer has a larger magnetic moment and more frequently collides with the cell wall; therefore, it gets stronger perturbations and a higher spin conversion efficiency, resulting in the surface-enhanced direct nuclear-spin conversion in the ortho isomer possibly being more effective (Supplementary Note 1). The rate *γ*_mm_=*γ*_op_=*ap*/(*b*+*p*^2^)+*cp* is a pressure-dependent term. It is obtained from the model of quantum relaxation and expresses the ortho–para conversion rate of gaseous CH_3_OH. From the experimental observations by an *A*-line (Case 1) and an *E*-line (Case 2), we have obtained the ortho–para conversion rate of CH_3_OH and have shown in Table 1 that the ortho–para conversion parameters in Cases 1 and 2 agree well within the experimental errors, suggesting a spin conversion mechanism in CH_3_OH is via quantum relaxation. Accordingly, we have found that the energy gaps between a number of the mixed pairs of the *A*- and *E*-levels of CH_3_OH are close to Γ_*αα*′_/2*π*=*Cp* for *p* in 0.3–2 Torr with *C*=20 MHz Torr^−1^ (Supplementary Table 1), which can be strongly mixed by the weak nuclear-spin-dependent intramolecular hyperfine interactions of CH_3_OH. Mixing and interruption of this mixing by molecule–molecule collisions within the bulk gas result in conversions of NSIs^14^.

In this work, we have studied on an important subject of NSIs of molecules, which has fundamental significance in a wide variety of scientific disciplines. This work is the first experiment performed at ambient temperature on NSIs of CH_3_OH, which are combined directly with states involving the torsional symmetry of the *A*- and *E*-species; therefore, it adds a new molecule of CH_3_OH to the short list where interconversions of NSIs of molecules in the gas phase have been observed. We have experimentally determined the pressure dependence of nuclear-spin conversion rate of CH_3_OH, which is important and very desirable to many scientific researches in Physics and Chemistry, and to practical applications in Astronomy and Astrophysics, and is well explained by the same model of quantum relaxation as that used for explanations of the linear pressure dependences of the conversion rates of NSIs of CH_3_F, ^13^CH_3_F, ^13^CCH_4_, C_2_H_4_ and H_2_CO. In contrast and of importance, in this work the quantum relaxation model is applied to explain the decreasing of nuclear-spin conversion rate with increasing pressure for CH_3_OH. Such a quantitative interpretation reveals the existence of weak intramolecular hyperfine interactions in CH_3_OH and suggests that they should be extended to include interactions of nuclear spin–spin, nuclear spin–rotation and nuclear spin–torsion in a modified model of quantum relaxation for torsional molecules. In this work, as in our previous work done for C_2_H_4_ in ref. 9, we have taken LID as a tool for separation of the ortho and para isomers of CH_3_OH. However, here we have used the new property of the LID technique, which modifies the CCS of the excited molecules of the ortho or para isomer of CH_3_OH being smaller than that of the ground-state molecules, which is in opposite to that used for C_2_H_4_ in ref. 9. The applications of these two properties of the LID technique demonstrated in our two works together make it a sensitive tool to create a non-equilibrium isomeric mixture for study of the nuclear-spin conversion of gaseous molecules.

In conclusion, using a CO_2_ laser we have produced non-equilibrium populations between the ortho- and para-CH_3_OH isomers by velocity-selective excitation. The separation observations and signals clearly show that the enrichment or depletion of spin isomers of CH_3_OH follows an unusual LID process in which the CCS of the excited isomer molecules is smaller than that of their ground-state counterparts (see also Supplementary Table 2). Our spin conversion observations provide experimental evidence for the existence of weak nuclear-spin-dependent intramolecular hyperfine interactions in CH_3_OH and reveal that the conversion is inhibited with increasing collision rates at higher pressures, demonstrating the quantum Zeno effect induced by collisions in a free molecule. The results provide basic experimental information for application to nuclear-magnetic resonance signal enhancement^10^ and for further improvements in existing knowledge about the dynamics of separation and conversion of NSIs of torsion-type molecules in various scientific disciplines. It will also be interesting as a potential application of the conversion rates observed here for CH_3_OH in the microscopic world to extend them to the macroscopic universe, notably in interstellar space^19^, to explore the formations and evolutions of stars and planets.

## Methods

### The three-period signal recording method

In our experiment, we use a dual-beam differential absorption method to sensitively probe the spin-isomer compositions by a liquid-N_2_-cooled HgCdTe detector. Two 20-cm-long detection cells (test cell and reference cell) are set side-by-side in parallel. The test cell is connected to the front end of the 1-m-long separation cell via a glass valve-*T* and the reference cell is kept at the equilibrium composition. Apparently, the output of the detector should be zero when the separation laser is blocked and not introduced to the separation cell, giving a zero-difference baseline in the recorded absorption signals in Stage 1. The first signal period is usually set at ∼30 s. After 30 s, the separation laser was suddenly unblocked and thus the high-power laser beam was focused through the separation cell. This is a process involving LID effect, which causes the drift of one isomer species and produces a non-equilibrium isomeric mixture near the front end of the separation cell and in the test cell through the valve-*T*. The optimum time for this period, which corresponds to the second signal period Stage 2, usually takes ∼60 s before the valve-*T* is closed to get an isolated test cell from the separation cell. At the 90th second, the valve-*T* was closed. The subsequent conversion signals between ortho and para isomers were then observed by the HgCdTe detector from the transmissions of the two detection cells, processed by a lock-in amplifier at a time constant of 0.3 s, and recorded by a computer.

### Derivation of equation *γ*_op_≈*ap*/(*b*+*p* ^2^)+*cp*

From equation (1), we have


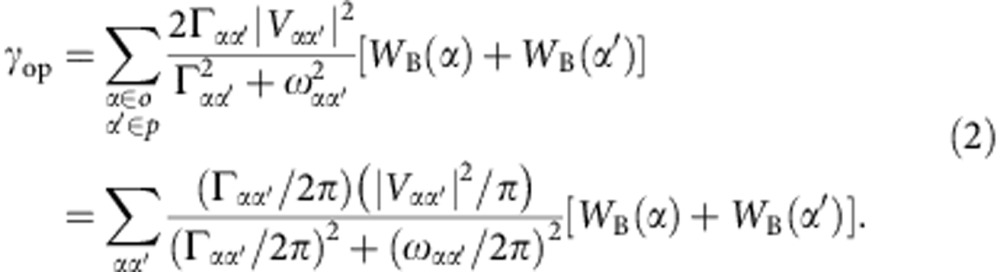


Taking the approximation of Γ_*αα*′_/2*π*=*Cp* with pressure *p* and *C*=20 MHz Torr^−1^ (refs 22, 27) for all transition levels, we can rewrite equation (2) as


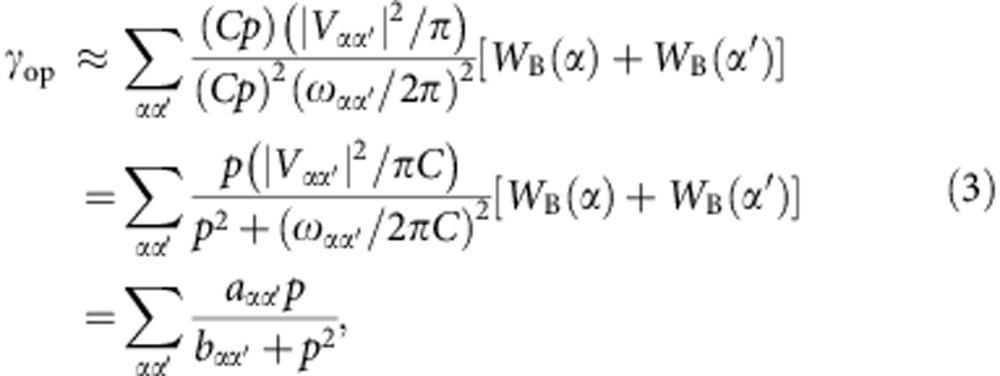


where *a*_*αα*′_=(|*V*_*αα*′_|^2^/*πC*)[*W*_B_(*α*)+*W*_B_(*α'*)], *b*_*αα*′_=(*ω*_*αα*′_/2*πC*)^2^.

In terms of energy gaps, one can classify all pairs of the *A*- and *E*-levels with the variable *α*∈*o*,*α*′∈ *p* into the variable category *m*∈*o*, *m*′∈ *p* with *b*_*mm*′_∼*p*^2^ and *n*∈*o*, *n*′∈ *p* with *b*_*nn*′_>>*p*^2^. Thus, from equation (3), we have


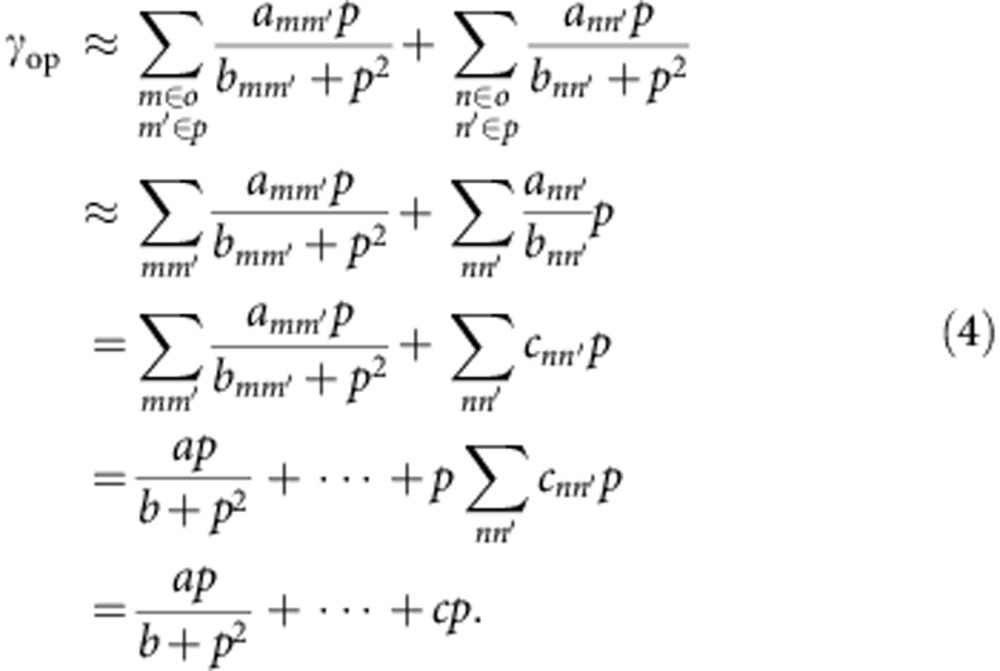


Suppose parameters *a* and *b* are related to the level pair with the closest energy gap, the rate of the conversion contributed from weak nuclear-spin-dependent intramolecular hyperfine interactions can then be approximately expressed as


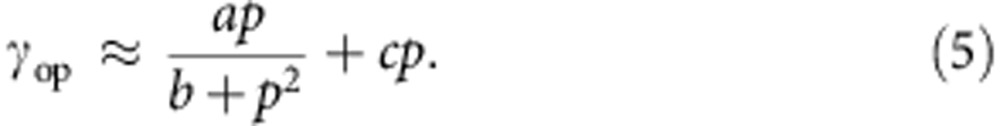


Suppose a pressure-independent term *d* approximately expresses the induced rate of spin conversion by the interactions of isomer molecules with the wall of the test cell in Stage 3, the observed conversion rate *γ* in our experiment can be written as


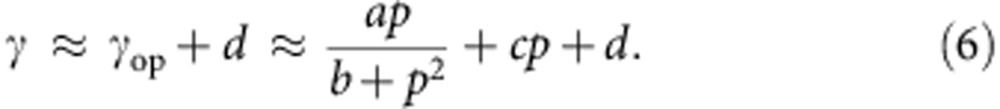


## Author contributions

Z.-D.S. contributed to the experimental setup, conducted measurements, analysed the data and wrote the manuscript. M.G. performed the calculations and data analyses. Y.Z. carried out results interpretation. All authors discussed the results and the manuscript.

## Additional information

**How to cite this article:** Sun, Z.-D. *et al.* Separation and conversion dynamics of nuclear-spin isomers of gaseous methanol. *Nat. Commun.* 6:6877 doi: 10.1038/ncomms7877 (2015).

## Supplementary Material

Supplementary Information Supplementary Tables 1-2, Supplementary Note 1 and Supplementary References

## Figures and Tables

**Table 1:** Experimental schemes and the determined parameters in *γ*_op_≈*ap/*(*b*+*p* ^2^)+c*p* at 300 K.

**Case and number**	**CH**_**3**_**OH transition**	**NSI**	**Laser line**	Δ***f*****(MHz)**	***a*** **(10**^**−2**^**) (s**^**−1**^ **Torr)**	***b*** **(10**^**−2**^**) (Torr**^**2**^**)**	***c*** **(10**^**−2**^**) (s**^**−1**^ **Torr**^**−1**^**)**
Separation	*R*(*A* 0 0, 10)	Ortho	9*P*16	67			
Probe							
1	*R*(*A* 0 5, 13)	Ortho	9*P*12	107	1.39±0.12	3.2±1.3	0.72±0.08
2	*P*(*E* 0 1, 5)	Para	9*P*42	−133	1.57±0.23	5.2±2.7	0.63±0.12
							
Average of Cases 1 and 2					1.48±0.18	4.2±2.0	0.68±0.10

The CH_3_OH transition *R/P*(*σ υ*_t_ *K*, *J*) is from the ground state to the CO-stretching excited state. Here, *υ*_t_ is the torsional quantum number, and *J* and *K* are quantum numbers of the rotational angular momentum and its projection along the axis of the CH_3_ group, respectively. Frequency offset Δ*f* denotes the CH_3_OH transition centre frequency minus the CO_2_ laser centre frequency.

## References

[b1] Landau L. D., Lifshitz E. M. Quantum Mechanics 3, Pergamon (1981).

[b2] Farkas A. Orthohydrogen, Parahydrogen, and Heavy Hydrogen Cambridge Univ. Press (1935).

[b3] Chapovsky P. L. Conversion of nuclear spin modifications of CH_3_F molecules in the gaseous phase. JETP 70, 895–901 (1990).

[b4] Nagels B., Schuurman M., Chapovsky P. L. & Hermans L. J. F. Nuclear spin conversion in molecules: Experiments on ^13^CH_3_F support a mixing-of-states model. Phys. Rev. A 54, 2050–2055 (1996). 9913693 10.1103/physreva.54.2050

[b5] Nagels B., Hermans L. J. F. & Chapovsky P. L. Quantum Zeno effect induced by collisions. Phys. Rev. Lett. 79, 3097–3100 (1997).

[b6] Cacciani P., Cosléou J., Herlemont F., Khelkhal M. & Lecointre J. Nuclear spin conversion in the gaseous phase in the presence of a static electric field: Intramolecular magnetic interactions and the role of collisions. Phys. Rev. A. 69, 1–8 (2004).

[b7] Peters G. & Schramm B. Nuclear spin state relaxation in formaldehyde: Dependence of the rate constant on pressure. Chem. Phys. Lett. 302, 181–186 (1999).

[b8] Chapovsky P. L., Cosléou J., Herlemont F., Khelkhal M. & Legrand J. Separation and conversion of nuclear spin isomers of ethylene. Chem. Phys. Lett. 322, 424–428 (2000).

[b9] Sun Z.-D., Takagi K. & Matsushima F. Separation and conversion dynamics of four nuclear spin isomers of ethylene. Science 310, 1938–1941 (2005). 16373570 10.1126/science.1120037

[b10] Zhivonitko V. V., Kovtunov K. V., Chapovsky P. L. & Koptyug I. V. Nuclear spin isomers of ethylene: Enrichment by chemical synthesis and application for NMR signal enhancement. Angew. Chem. Int. Ed. 52, 13251–13255 (2013). 10.1002/anie.201307389 24127422

[b11] Kravchuk T. *et al.* A magnetically focused molecular beam of ortho-water. Science 331, 319–321 (2011). 21252342 10.1126/science.1200433

[b12] Horke D. A., Chang Y.-P., Długołęcki K. & Küpper J. Separating para and ortho water. Angew. Chem. Int. Ed. 53, 11965–11968 (2014). 10.1002/anie.201405986 25196938

[b13] Gel'mukhanov F. Kh. & Shalagin A. M. Light-induced diffusion of gases. JETP Lett. 29, 711–713 (1979).

[b14] Chapovsky P. L. CH_3_F spin-modification conversion induced by nuclear magnetic dipole-dipole interactions. Phys. Rev. A 43, 3624–3630 (1991). 9905450 10.1103/physreva.43.3624

[b15] Dickens J. E. & Irvine W. M. The formaldehyde ortho/para ratio as a probe of dark cloud chemistry and evolution. Astrophys. J. 518, 733–739 (1999). 11543064 10.1086/307317

[b16] Kawakita H. *et al.* The spin temperature of NH_3_ in Comet C/1999S4 (LINEAR). Science 294, 1089–1091 (2001). 11691989 10.1126/science.1064339

[b17] Dello Russo N. *et al.* Tennyson, production rates, rotational temperatures and spin temperatures in comets C/1999 H1 (Lee), C/1999 S4, and C/2001 A2. Astrophys. J. 621, 537–544 (2005).

[b18] Hogerheijde M. R. *et al.* Detection of the water reservoir in a forming planetary system. Science 334, 338–340 (2011). 22021851 10.1126/science.1208931

[b19] Tielens A. G. G.. M. The molecular universe. Rev. Mod. Phys. 85, 1021–1081 (2013).

[b20] Ball J. A., Gottlieb C. A., Lilley A. E. & Radford H. E. Detection of methyl alcohol in Sagittarius. Astrophys. J. Lett. 162, L203–L210 (1970).

[b21] Batrla W., Mathews H. E., Menten K. M. & Walmsley C. M. Detection of strong methanol masers towards galactic HII regions. Nature 326, 49–51 (1987).

[b22] Moruzzi G., Winnewiser B. P., Winnewiser M., Mukhopadhyay I. & Strumia F. Microwave, Infrared and Laser Transitions of Methanol: Atlas of Assigned Lines from 0 to 1258 cm^−1^ CRC Press (1995).

[b23] Lees R. M. Giant *K* doubling in the infrared spectrum of CH_3_OH: A sensitive probe for CH_3_-rock/CO-stretch/OH-bend vibrational coupling. Phys. Rev. Lett. 75, 3645–3648 (1995). 10059691 10.1103/PhysRevLett.75.3645

[b24] Lees R. M., Xu L.-H., Johns J. W. C., Winnewisser B. P. & Lock M. Rotation–torsion–vibration term-value mapping for CH_3_OH: Torsion-mediated doorways and corridors for intermode population transfer. J. Mol. Spectrosc. 243, 168–181 (2007).

[b25] Hermans L. J. F. Light-induced kinetic effects in molecular gases. Int. Rev. Phys. Chem. 11, 289–315 (1992).

[b26] Curl R. F. Jr., Kasper J. V. V. & Pitzer K. S. Nuclear spin state equilibration through nonmagnetic collisions. J. Chem. Phys. 46, 3220–3228 (1967).

[b27] Lees R. M. & Haque S. S. Microwave double resonance study of collision induced population transfer between levels of interstellar methanol lines. Can. J. Phys. 52, 2250–2271 (1974).

[b28] Misra B. & Sudarshan E. C. G. The Zeno's paradox in quantum theory. J. Math. Phys. 18, 756–763 (1977).

