# Supplementary material for: Separation and conversion dynamics of nuclear-spin isomers of gaseous methanol
Source: Nat Commun. 2015 Apr 16;6:6877. doi: 10.1038/ncomms7877 (PMC4411283; doi:10.1038/ncomms7877)
Supplement: Supplementary Information — Supplementary Tables 1-2, Supplementary Note 1 and Supplementary References [file ncomms7877-s1.pdf]

Supplementary Table 1| The calculated energy gaps $\omega_{\alpha\alpha'}/2\pi$ (less than 47 MHz) with the estimated uncertainties between mixed pairs of the *A*- and *E*-levels of CH_3OH allowed by the selection rules $|\Delta J| \leq 2$, $|\Delta K| \leq 2$. The quantum number v denotes gr, co, oh, and ab for the ground, CO-stretching, OH-bending, and asymmetric CH_3-bending vibrational modes, respectively. The *A*- and *E*-level term values are from ref. 1 and the supplementary material of ref. 2. The reference energy of the lowest *A*-level is 127.97549 cm^{-1} in the (*A* gr 0 0, 0) and that of the lowest *E*-level is 133.46494 cm^{-1} in the (*E* gr 0 -1, 1).

Level pair number	Mixed *A*-level (A v $\upsilon_t K^{\pm}$, J)	Term value of *A*-level (cm^{-1})	Mixed *E*-level (E v $\upsilon_t K$, J)	Term value of *E*-level (cm^{-1})	Energy gaps $\omega_{\alpha\alpha'}/2\pi$ (MHz)
1	(*A* gr 0 4^-, 24)	667.48812	(*E* gr 0 2, 25)	667.48968	47 ± 8
2	(*A* gr 3 6, 28)	1611.91988	(*E* gr 2 8, 28)	1611.92075	26 ± 8
3	(*A* gr 4 0, 26)	1737.18567	(*E* co 0 2, 26)	1737.18680	34 ± 8
4	(*A* co 2 3, 17)	1862.34108	(*E* ab 0 4, 15)	1862.34034	22 ± 10
5	(*A* gr 3 1^+, 34)	1887.75088	(*E* gr 3 0, 35)	1887.75049	12 ± 10

Supplementary Table 2| The assignments, line intensities, and the transition frequencies of the CH_3OH lines in the frequency range near the CO_2 laser line of 9*P*16 (refs 1,3). Frequency offset Δ*f* denotes the CH_3OH transition frequency *f* minus the center frequency of 9*P*16 CO_2 laser line f_0(9*P*16): $\Delta f = f - f_0(9P16)$.

CH_3OH transition $R(\sigma\ \upsilon_t\ K^{\pm}, J)$	Line intensity (10^{3} cm^{-2} atm^{-1})	Transition frequency f (cm^{-1})	Δ*f* (MHz)
R(*E* 0 -7, 10)	69	1050.39196	-1479
$(A\ 1\ 0,\ 16)^{gr} \rightarrow (A\ 1\ 2^{+},\ 17)^{ro}$	27	1050.43934	-58
9*P*16 CO_2 laser line		1050.44128185	0
R(*A* 0 0, 10)	231	1050.44352	+67
R(*E* 1 -6, 10)	35	1050.45175	+314
R(*A* 0 9, 10)	46	1050.47309	+954

As shown in Supplementary Table 2, the *R*(*A* 0 0, 10) line is about 9 times stronger than the $(A\ 1\ 0,\ 16)^{gr} \rightarrow (A\ 1\ 2^{+},\ 17)^{ro}$ line in spectral intensity. Therefore in our experiment, we used the 9*P*16 CO_2 laser line and tuned the laser output frequency at f_0(9*P*16) + 20 MHz to resonance with the *R*(*A* 0 0, 10) line for the separation observation of the nuclear-spin isomers of CH_3OH; we found that the collision cross-section of the excited isomer molecules of CH_3OH is smaller than that of their ground-state counterparts. The 9*P*16 CO_2 laser line was also used previously for isotope separation of CH_3OH in mixtures with a much higher concentration of CH_3OH as absorbing species than that of its isotopes as buffer species[4], but the opposite result to ours about the collision cross-section in vibrationally excited state of CH_3OH was obtained due to the mistaken of the position of the 9*P*16 CO_2 laser line relative to the methanol absorption line.

Supplementary Note 1:

About the origins of the term d in the expression of $\gamma \approx \gamma_{op} + d \approx ap/(b + p^2) + cp + d$

It is speculated that some subtle factors could be the origins of the term *d*. We tentatively attribute to the following as one of these factors.

In Stage 2, a group of velocity-selective ortho isomers are heated and excited by the separation laser, which causes the corresponding increases in their thermal velocities and fast transports of them into the test cell by the unusual LID effect via the valve-*T* from the separation cell. The rapid influxes of the hot and enriched ortho isomer together with its collision partner are blocked by the wall surface of the test cell where the CH_3OH adsorption is in a dynamically balanced state in Stage 1, giving rise to the head-on collisions of these hotter isomers on the wall of the test cell. Therefore around the collision places, enhanced multilayer chemisorptions and desorptions of the CH_3OH isomers may occur, the breaking and reforming of the highly active O-H bond and the CH_3O^- (methoxy) radical of CH_3OH as well as unpaired electrons may exist. Consequently, a "dune"-like magnetic surface over there may appear and an inhomogeneous magnetic field within the part volume of the test cell may be induced. It may be able, in Stage 3 when the test cell has been isolated, to exert magnetic forces on protons of the H atoms of the ortho and para isomers in the bulk gas to induce spatial variations of the precession angles of their nuclear spins, which could be strong enough to flip the hydrogen spins of the isomers when the isomers move very close to or bump against the induced magnetic surface of the isolated test cell. For one species, the induced-rate of spin conversion by such an interaction of isomer molecules with the wall of the test cell in Stage 3 might be approximately taken as a pressure-independent term. For example, although more isomer molecules will submit to the magnetic field in the isolated test cell at a higher pressure, the magnetic field gradients will be smaller due to less amount of the enhanced net adsorptions of the isomer molecules on the cell wall than that at a lower pressure. Therefore, the whole surface-molecule interaction and the interaction efficiency to the spin conversion of the isomer may be approximately close to each other at various pressures. For different species, comparing to the para isomer, the enriched ortho isomer has a larger magnetic moment and more frequently collides with the cell wall, therefore it gets stronger perturbations and a higher spin conversion efficiency, resulting in the surface-enhanced direct nuclear spin conversion in the ortho isomer possibly being more effective.

Supplementary References

1. Moruzzi, G., Winnewiser, B. P., Winnewiser, M., Mukhopadhyay, I. & Strumia, F. *Microwave, Infrared and Laser Transitions of Methanol: Atlas of Assigned Lines from 0 to 1258 cm^{-1}* (CRC Press, Boca Raton, 1995).

2. Lees, R. M., Xu, L.-H., Johns, J. W. C., Winnewisser, B. P. & Lock, M. Rotation–torsion–vibration term-value mapping for CH_3OH: Torsion-mediated doorways and corridors for intermode population transfer. *J. Mol. Spectrosc*. **243**, 168–181 (2007).

3. Lees, R. M. Giant *K* doubling in the infrared spectrum of CH_3OH: A sensitive probe for CH_3-rock/CO-stretch/OH-bend vibrational coupling. *Phys. Rev. Lett.* **75**, 3645–3648 (1995).

4. Bakarev, A. E., Ishikaev, S. M. & Chapovsky, P. L. Inverse light-induced drift of methanol and CH_3F molecules. *Sov. J. Quantum Electron*. **18**, 890–893 (1988).
